# Treatment of Pellagra

**Published:** 1909-10

**Authors:** 


### Treatment of Pellagra.

MUCH interest has been aroused during the last two years in the subject of pellagra. A study of the disease in the United States has thus far shown that it is widely distributed throughout the south, and present in some localities in the north. The question of prognosis and treatment is naturally therefore, one of much interest. Dr. C. H. Lavinder, of the Public Health and Marine Hospital Service, who for more than a year has been devoting his time to a study of the disease, has in a recent article[1] given a brief review of the subject.

He states that the prognosis must invariably be considered as grave, and that complete recovery can seldom be assured. Reliable statistics on the subject in the United States are practically limited to asylum cases, and give a mortality of 67%. It must be borne in mind, however, that asylum cases are undoubtedly more advanced and hopeless ones, and for that reason will give a mortality much above the average. Lombroso gives statistics of hospital cases in Italy in 1883 and 1884, showing a mortality of 13%, whereas Wollenberg gives Italian statistics for 1905, showing a mortality of but little over 4%. The disease resembles tuberculosis, both in that it is an insidious and chronic condition, and that much depends upon early diagnosis and treatment, prognosis of early cases being far better than advanced ones. The importance of this is apparent when it is considered that the disease is an intoxication, and that it is probably associated with diseased corn or corn products used as food.

Predisposition is believed to be an important factor in this disease. Lowered physical resistance, mental worry, insufficient food, bad housing and alcoholism are supposed to render one more susceptible.

In Italy laws have been passed regulating the use and storing of corn and its derivatives, institutions have been established for the care and treatment of Pellagrins, improved agricultural methods are encouraged, and assistance is given to the sick in many ways by the Government.[2]

In the treatment of cases Lombroso recommends a liberal diet; in some cases he uses baths and cold douches, believing them to be of benefit in certain cases with nerve and skin manifestations; he has found arsenic a valuable remedy, and sodium chloride of service. Some authors have reported good results from the use of the newer arsenical preparations atoxyl and soamin. Transfusion of blood from cured cases to the sick has been tried, and may prove of value.

1. Public Health Reports, September 10, 1909. Copies of this article can be obtained by making request to the Surgeon-General, Public Health and Marine Hospital Service, Washington, D. C.

2. Public Health Reports, July 23, 1909, pp. 1053-1054.

CHICAGO'S health commissioner, Dr. W. A. Evans, according to the *Tribune* has branched out into an inventor—an expert on homemade ice boxes. He has a plan for constructing a refrigerator for 25 cents which, he asserts, can be operated for about two cents a day. In a bulletin addressed to the poor of Chicago, Dr. Evans says: "Thousands of babies die each year simply because they are fed bad milk; especially is this true in hot weather. Clean milk requires only ice to preserve it, but very few poor families possess a refrigerator. Secure an ordinary wooden box, 13x18 inches, with a depth of 11½ inches, from your grocer. In the bottom of the box place a substantial layer of sawdust. In this set a tin pail or can, eight inches in diameter and high enough to hold a quart bottle of milk. Care should be taken that the pail rests on sawdust—not on the wood bottom of the box. Around the pail place a cylinder of tin a little larger than the pail, then pack sawdust about the cylinder—not between pail and cylinder—up to the top of the cylinder. On the cover of the box nail about fifty layers of newspaper. Set the milk bottle in the pail an pack broken ice about the bottle."

---

ELSEWHERE we publish an appeal to the medical profession of the west and south, relating to the preservation of historical and biographical records. An association has been formed, of which Dr. Charles A. L. Reed, is president, and the headquarters of which is at Cincinnati. The association wishes to obtain journals, books, pamphlets, manuscripts, autographs, society proceedings, reports of hospitals, catalogues and announcements of Medical schools, biographies and portraits of physicians, curios, and the like. While this appeal is made in particular to physicians of the west and south, it would be well it seems to us, for similar organisations to rise up in the north and east. The object is a good one. Contributions in this instance should be sent prepaid to Dr. A. G. Drury, Librarian, 710 W. Eighth street, Cincinnati, Ohio.

---

THE *Military Surgeon* of July, 1909, publishes a most interesting case of recovery of penetrating injury of the brain, in which a man had both temples penetrated by a 32-calibre bullet, was treated by Dr. W. Armistead Gills, of Richmond, Va. It is the only case of its character recorded and is especially interesting for the reason that the functions of the frontal lobes are not disturbed, and none of the vessels or nerves seem to have suffered, the only apparent trouble being a disturbance of the olfactory tract. Brain substance was present upon the cheek. Dr. Gills is an ex-student officer of the Army Medical School, and from his instruction in military surgery, has presented his case in a very comprehensive fashion, describing the velocity of the missile and the kind of wounds produced by such bullets. The patient today, is every way in a normal condition.

---

So widespread has interest in the strange malady, pellagra, become among medical authorities and others throughout the country that Surgeon General Wyman of the Public Health and Marine Hospital Service, according to a recent Washington despatch, has decided to issue a weekly bulletin dealing exclusively with the developments of that disease. This step has been taken as a result of requests from practically all the state boards of health, which are watching the progress of the disease, particularly in the south, with much concern.

The bulletin will not only show the prevalence of pellagra, but will indicate the distribution of the disease. The statistics for the publication will be furnished by the medical authorities of the state and territories. This information, it is believed, will be of great aid in determining the cause of pellagra, and help materially in the efforts of the government to check its progress.

Dr. Wyman was greatly interested to learn today of the action of the Tennessee Board of Health in quarantining against the disease, it being held that the malady is communicable. While declining to go on record as believing that pellagra is not communicable, he said he had observed nothing in the disease to show that it is contagious. The consensus of opinion among medical authorities in Italy, where the disease has existed for a long time, Dr. Wyman said, is that it is non-contagious. This view is also held by Dr. C. H. Lavinder, of the Public Health and Marine Hospital Service, who is devoting his entire time to studying the disease. Elsewhere, we publish editorially some comment by Dr. Lávinder on the peculiar malady.

